# Hepatitis C Virus Non-Structural Protein 3 Interacts with Cytosolic 5′(3′)-Deoxyribonucleotidase and Partially Inhibits Its Activity

**DOI:** 10.1371/journal.pone.0068736

**Published:** 2013-07-09

**Authors:** Chiu-Ping Fang, Zhi-Cheng Li, Chee-Hing Yang, Ju-Chien Cheng, Yung-Ju Yeh, Tsai-Hsia Sun, Hui-Chun Li, Yue-Li Juang, Shih-Yen Lo

**Affiliations:** 1 Department of Laboratory Medicine and Biotechnology, Tzu Chi University, Hualien, Taiwan; 2 Department of Medical Laboratory Science and Biotechnology, China Medical University, Taichung, Taiwan; 3 Department of Biochemistry, Tzu Chi University, Hualien, Taiwan; 4 Department of Microbiology, Tzu Chi University, Hualien, Taiwan; 5 Department of Laboratory Medicine, Buddhist Tzu Chi General Hospital, Hualien, Taiwan; Institut National de la Santé et de la Recherche Médicale, France

## Abstract

Infection with hepatitis C virus (HCV) is etiologically involved in liver cirrhosis, hepatocellular carcinoma and B-cell lymphomas. It has been demonstrated previously that HCV non-structural protein 3 (NS3) is involved in cell transformation. In this study, a yeast two-hybrid screening experiment was conducted to identify cellular proteins interacting with HCV NS3 protein. Cytosolic 5′(3′)-deoxyribonucleotidase (cdN, dNT-1) was found to interact with HCV NS3 protein. Binding domains of HCV NS3 and cellular cdN proteins were also determined using the yeast two-hybrid system. Interactions between HCV NS3 and cdN proteins were further demonstrated by co-immunoprecipitation and confocal analysis in cultured cells. The cellular cdN activity was partially repressed by NS3 protein in both the transiently-transfected and the stably-transfected systems. Furthermore, HCV partially repressed the cdN activity while had no effect on its protein expression in the systems of HCV sub-genomic replicons and infectious HCV virions. Deoxyribonucleotidases are present in most mammalian cells and involve in the regulation of intracellular deoxyribonucleotides pools by substrate cycles. Control of DNA precursor concentration is essential for the maintenance of genetic stability. Reduction of cdN activity would result in the imbalance of DNA precursor concentrations. Thus, our results suggested that HCV partially reduced the cdN activity via its NS3 protein and this may in turn cause diseases.

## Introduction

Hepatitis C virus (HCV) is a major cause of chronic hepatitis, liver cirrhosis, and hepatocellular carcinoma [1]. Infection with HCV is also etiologically involved in the development of B-cell lymphomas [2]. This virus belongs to the genus *Hepacivirus* in the family *Flaviviridae*. The HCV genome is a single, positive-stranded RNA with a nucleotide length of about 9.6 kb. It encodes a polyprotein precursor of approximately 3,000 amino acids. This polyprotein precursor is processed by host and viral proteases into at least 10 different proteins, which are arranged in the order of NH2-C-E1-E2-p7-NS2-NS3-NS4A-NS4B-NS5A-NS5B-COOH. C, E1, and E2 are structural proteins while NS2-NS5B and perhaps also p7 are non-structural proteins. The release of C, E1, E2 and, p7 from the polyprotein is mediated by the cellular signal peptidase located in the endoplasmic reticulum, whereas the cleavages between NS2-NS5B are mediated by viral NS2/3 and NS3/4A proteases. NS3 protein contains a serine protease activity within its N-terminal 180 residues and NTPase and helicase activities in the C-terminus (for a review, [3]). Molecular mechanisms regarding HCV pathogenesis are not well understood. It has been demonstrated that HCV NS3 protein is involved in cell transformation [4], [5]. To further understand the functions of the HCV NS3 protein, we have conducted a yeast two-hybrid screening experiment to identify the cellular proteins interacting with HCV NS3 protein. Our results indicated that the cytosolic 5′(3′)-deoxyribonucleotidase (cdN, dNT-1) interacts with HCV NS3 protein [6], [7]. We further demonstrated that this interaction can result in the partial repression of the cdN activity.

## Materials and Methods

### Plasmid Construction

The expression plasmid for HCV NS3 protein used in this study was derived from the plasmid p90/HCV FL-long pU (GI: 2316097) which contains the full-length sequence of the HCV-H isolate. To isolate the cDNA fragment that contains the NS3/4A protein coding sequence, polymerase chain reactions (PCR) using primers (5′CGGGATCCGCGCCCATCACGGCGTAC 3′and 5′GCTCTAGACTATTAGCACTCTTCCATCTC3′) were performed. After PCR, the DNA fragment was digested with restriction enzymes (BamHI/XbaI) and inserted into the pcDNA3-myc vector for transient expression in mammalian cells [8]. To clone the DNA fragment encoding HCV NS3 protein (full-length, from a.a. 1 to 631) for yeast two-hybrid screening, oligonucleotide primers (5′GGAATTCGCGCCCATCACGGCG3′and 5′GCTCTAGACTATTACGTGACGACCTCCAG3′) were used to perform PCR. After PCR, the DNA fragment was treated with T4 polynucleotide kinase, digested with the restriction enzyme EcoRI, and cloned into the pBDGal4 Cam (Stratagene, USA) expression vector, which had been linearized with EcoRI and SmaI. Similar approaches were used to clone the DNA fragment encoding HCV NS3 protease domain (from a.a. 1 to a.a. 208) for yeast two-hybrid screening experiments except the oligonucleotide (5′GCTCTAGATTAGCTGCCGGTGGGAGC3′) was used as the reverse primer to perform PCR. The oligonucleotide primers (5′GGAATTCGTGGCCCACCTGCATG3′and 5′GCTCTAGATTACTCGGCGGGCGTGAG3′) were used to clone HCV NS3 protein helicase domain (from a.a. 199 to a.a. 508) for yeast two-hybrid screening.

To construct the expression plasmids of various recombinant cdN proteins, DNA fragments were amplified by the PCR from the 3′-UTR of RBaK cDNA which shares the identical sequences with the cdN coding region but without the initiation codon [9]. To isolate the cDNA fragment that contains the cdN protein coding sequence, PCR reactions using primers (5′CGGAATTCATGGCGCGGAGCGTGCGC 3′and 5′GCTCTAGATTCCCGCTGCGCAGCTCC3′) were performed. After PCR, the DNA fragment was digested with restriction enzymes (EcoRI/XbaI) and inserted into the pcDNA3.1-V5-His A (Invitrogen, USA) vector for transient expression in mammalian cells. To clone the full-length cdN DNA fragment for yeast two-hybrid screening (a.a. 1–201), primers (5′CCGAATTCGCATGGCGCGGAGCGTGCGC3′and 5′CCGCTCGAGTCATTCCCGCTGCGCAGC3′) were used to perform PCR. After PCR, the DNA fragment was digested with restriction enzymes (EcoRI and XhoI) and cloned into the pACT2 (Clontech, USA) expression vector (linearized with EcoRI/XhoI). The same forward primer and a different reverse primer (5′CCGCTCGAGTTAGTACACACTGGCCAC3′) were used to clone the DNA fragment that contained the first 65 codons of the cdN sequence for yeast two-hybrid screening. The same reverse primer and a different forward primer (5′CCGAATTCGCGGTGAGAAGTACCGC3′) were used to clone the DNA fragment encoding the C-terminal 92 a.a. of cdN protein (a.a. 110-201). The oligonucleotide primers (5′CCGAATTCGCGAAGCCCCGGGCTTT3′and 5′CCGCTCGAGTTACACACAGTGGTGGTA3′) were used to clone the DNA fragment encoding cdN protein from a.a. 66 to a.a. 109.

To construct the expression plasmid for a.a. 846 to 1008 of ELKS-δ protein [10], the DNA fragment was amplified by PCR from the cDNA library of HuH7 cells using primers (5′CGGAATTCGCGTGGAGGAGTTACTGATGGC3′ and 5′CCGCTCGAGTCAGTCATGGCAGAGGGTTG3′). After amplification, the DNA fragment was digested with restriction enzymes (EcoRI and XhoI) and cloned into the pACT2 expression vector (linearized with EcoRI/XhoI).

All the expression plasmids were verified by sequencing.

### Yeast Two-hybrid Screening

The yeast two-hybrid system used for the screening was purchased from Clontech Laboratories (USA). The screening procedures were conducted following the manufacturer’s instructions and our previous procedures [11], [12]. The cDNA library used for this screening was a human fetal liver library, HL4029AH (Clontech).

### Cell Culture

HuH7 cells were cultured in Dulbecco’s modified Eagle’s medium (DMEM) containing 10% fetal bovine serum (FBS), 100 U/ml penicillin and 100 µg/ml streptomycin (Gibco, USA) [13]. HCV sub-genomic replicon cells were cultured in DMEM with 10% FBS, 100 U/ml penicillin, 100 µg/ml streptomycin and 400 µg/ml G418 [14]. All cultured cells were maintained at 37°C with 5% CO_2_.

### DNA Transfection

The ExGen 500 *in vitro* transfection reagent (Fermentas, USA) was used to transfect DNA (e.g., pcDNA3.1-cdN or pcDNA3-myc-NS3/4A) into HuH7 cells following the manufacturer’s instructions.

### Western Blotting Analysis

Our previous procedures were followed for Western blotting analysis [11], [12], [13]. The primary antibodies used for the analyses in this study were goat anti-cdN polyclonal antibody (Santa Cruz, USA), mouse anti-NS5A monoclonal antibody (Biodesign, USA), mouse anti-myc (4A6) monoclonal antibody (Upstate, USA), mouse anti-V5 monoclonal antibody (Serotec, USA), mouse anti-actin monoclonal antibody (Santa Cruz), mouse anti-NS3 monoclonal antibody (Santa Cruz), mouse anti-mdN monoclonal antibody (Abnova, USA) and rabbit anti-Erk2 polyclonal antibody (Santa Cruz).

### Immunoprecipitation and Western Blotting Analysis

3–5×10^6^ cells were seeded in a 100-mm culture dish. After overnight incubation, cells were transfected with 2 ug plasmid DNA using the ExGen 500 *in vitro* transfection reagent. At 48 hours after transfection, cells were lysed in 1 ml RIPA (50 mM Tris-HCl, pH 7.5, 300 mM NaCl, 4 mM EDTA, pH 8.0, 0.5% Trition-X 100, 0.1% SDS and 0.5% sodium deoxycholate). After centrifugation for five minutes, the supernatant was incubated with the anti-V5 antibody (1∶200–1∶500 dilution) at 4°C overnight. The antibody-antigen complex was pulled down with Pansorbin (Merck, USA). The samples were treated at 100°C in the sample buffer (67.5 mM Tris-HCl (pH 6.8), 5% 2-mercaptoethanol, 3% SDS, 0.1% bromophenol blue and 10% glycerol) for 10 minutes followed by gel electrophoresis and Western-blot to PVDF paper (Pall Corporation, USA). The alkaline phosphatase-conjugated anti-V5 antibody (Invitrogen) and peroxidase-conjugated anti-myc antibody (Upstate) were used as the antibodies for the analysis. In each experiment, 5% of cell lysates were used for protein expression analysis directly while 95% of cell lysates were used for the co-immunoprecipitation assay.

### Confocal Microscopy Analysis

About 2.5×10^5^ cells were seeded in each 35 mm culture dish. After overnight incubation, cells were transfected with 0.4 ug plasmid using the ExGen 500 *in vitro* transfection reagent. At 48 hours after transfection, cells were fixed with methanol/acetone (1∶1) at 0°C for 10 minutes, washed with the incubation buffer (0.05% NaN_3_, 0.02% saponin and 1% skim milk in PBS) twice for 2 minutes each, and then incubated with the mouse anti-myc antibody (1∶200 dilution). Cells were washed with PBS at room temperature for five minutes three times, and then incubated with the RITC–conjugated goat anti-mouse IgG antibody (1∶200 dilution) at 3°C for 30 minutes. Later, cells were stained with the FITC-conjugated anti-V5 antibody (1∶200 dilution) at 37°C for 30 minutes. Cells were washed three more times with PBS. DAPI (Merck) was used to stain the nucleus.

For the detection of endogenous HCV NS3 and cellular cdN proteins, HCV subgenomic RNA replicon cells were used [14]. Mouse anti-NS3 monoclonal antibody and Goat anti-cdN polyclonal antibody were used as the primary antibodies.

### RNAi Experiments

RNAi experiments and establishment of cells with stably expressed exogenous proteins were performed using the lentiviral expressing system (http://rnai.genmed.sinica.edu.tw), following the manufacturer’s instructions. RNAi reagents were obtained from the National RNAi Core Facility located at the Institute of Molecular Biology/Genomic Research Center, Academia Sinica, Taiwan.

### Assay for 5′ (3′)-deoxyribonucleotidase Activity

The assay for the 5′ (3′)-deoxyribonucleotidase activity in this study followed the condition described by a previous report [15]. The nucleotide [^3^H] dUMP (Moravek Biochemicals Inc., USA) was used as the substrate. Specific enzyme activity is nmol nucleoside formed per minute per milligram of protein. All assays were done in three independent experiments in duplicates with two different amounts of protein (0.2 and 0.8 ug). Results derived from two different amounts of protein are almost identical. Only results from 0.2 ug were presented in this study.

### Generation of Infectious HCVcc and Infectivity Assay

Infectious HCV particles (HCVcc) were obtained as described previously [16], [17], [18]. To generate infectious HCVcc, *in vitro*-transcribed genomic J6/JFH RNA was delivered into HuH7.5 cells by electroporation. The HCVcc was recovered from cell culture medium after passage for 2 weeks. The virus-containing supernatant was clarified by low-speed centrifugation, passed through a filter with the pore size of 0.45 µm, and concentrated by ultracentrifugation. For the infectivity assay, HuH7.5 cells were separated into 3.5 cm Petri dish at the density of 2×10^5^ cells. After 4 hrs, the cells were attached and 100 µl of HCVcc-containing supernatant (MOI = 0.1) was added to each dish and incubated for additional 72 hrs. The mock- and HCV-infected cell lysates were then harvested for Western blot assay to detect protein expression and for the analysis to detect 5′ (3′)-deoxyribonucleotidase activity.

## Results

### Identification of Cytosolic 5′ (3′)-deoxyribonucleotidase (cdN, dNT-1) as an Interactive Protein of HCV NS3 Protein by Yeast Two-hybrid Screening

**Figure 1:**
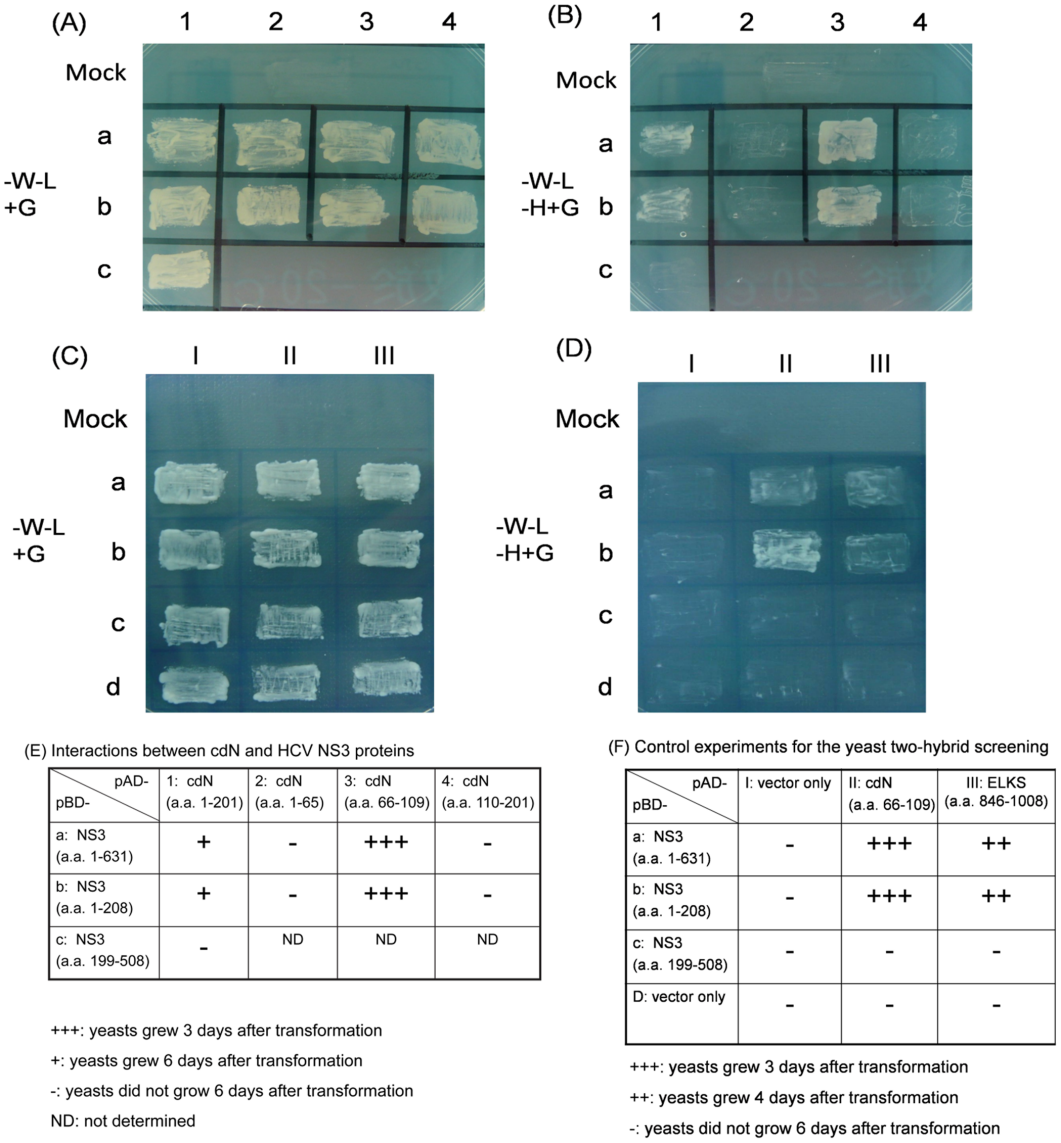
Interactions between HCV NS3 and cellular cdN proteins in yeast. Growth of yeasts that had been either mock-transfected or transfected with different combinations of plasmids as indicated; the transfected yeast cells were grown in YEPD without tryptophan and leucine (A and C), or YEPD without tryptophan, leucine and histidine (B and D). (E) Summarized results of (A) and (B): HCV NS3 protease domain interacts with cdN (a.a. 66-109). (F) Summarized results of (C) and (D).

HCV NS3 protein was used as the bait for yeast two-hybrid screening for the identification of cellular proteins that interact with NS3 protein. Only one cDNA clone, which encoded a truncated cdN protein without its N-terminal 44 amino acids, was found to interact with the NS3 protein out of a total of 1×10^6^ transformants. To further identify the interactive domains of cdN protein with NS3 protein, we also conducted deletion-mapping experiments. As shown in Fig. 1 (panels A and B), the middle region of cdN protein (a.a. 66-109) interacted with the protease domain of NS3 protein (Fig. 1E).

Several negative controls were included to assure the specificity of this assay, and a.a. 846 to 1008 of ELKS-δprotein (a domain known to interact with HCV NS3) [10] was served as the positive control (Fig. 1 C, D). Indeed, a.a. 846 to 1008 of ELKS-δprotein could interact with the NS3 protease domain and no non-specific interactions was observed in the negative controls (Fig. 1F).

### Physical Interactions between cdN and HCV NS3 Proteins in Cultured Cells

**Figure 2:**
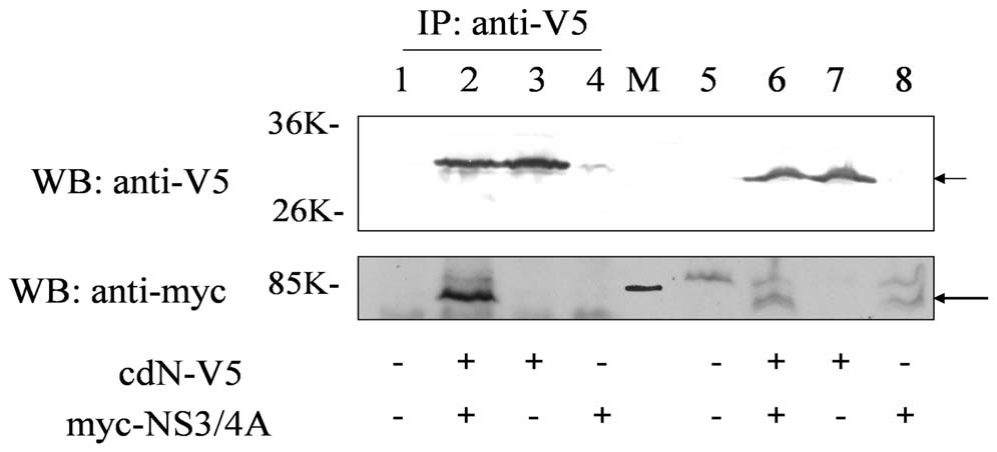
Co-immunoprecipitation experiments of HCV NS3/4A and cdN expressed in HuH7 cells. HuH7 cells were transfected with empty vector (lanes 1 and 5, 4 ug of DNA), with NS3/4A protein tagged with myc (lanes 4 and 8, 2 ug of myc-NS3/4A plus 2 ug of empty vector), with cdN tagged with V5 (lanes 3 and 7, 2 ug of cdN-V5 plus 2 ug of empty vector) or co-transfected with myc-tagged NS3/4A and V5-tagged cdN (lanes 2 and 6, 2 ug DNA of each). Cell lysates were directly analyzed by Western-blotting (lanes 5-8, 5% of total lysates) or immunoprecipitated with the anti-V5 antibody (lanes 1-4, 95% of total lysates) prior to Western-blotting using antibodies against the myc tag to detect NS3/4A protein (bottom panel) and against the V5 tag to detect cdN (upper panel). The cdN-V5 protein and the myc-NS3/4A protein were marked with two different arrows. The protein markers were loaded in the middle lane labeled as “M”.

To further test whether cdN and NS3 proteins could bind to each other in cells, we also performed the co-immunoprecipitation experiment. The myc-tagged full-length NS3/4A protein and the V5-tagged cdN protein were co-expressed in HuH7 cells by transient transfection. After transfection, cell lysates were immunoprecipitated with the anti-V5 antibody followed by Western blotting using the anti-myc antibody. As shown in Fig. 2, the myc-tagged NS3/4A protein could be immunoprecipitated by the anti-V5 antibody in the presence (lane 2), but not in the absence (lane 4), of the cdN protein. This result further confirmed that NS3 and cdN could bind to each other.

**Figure 3:**
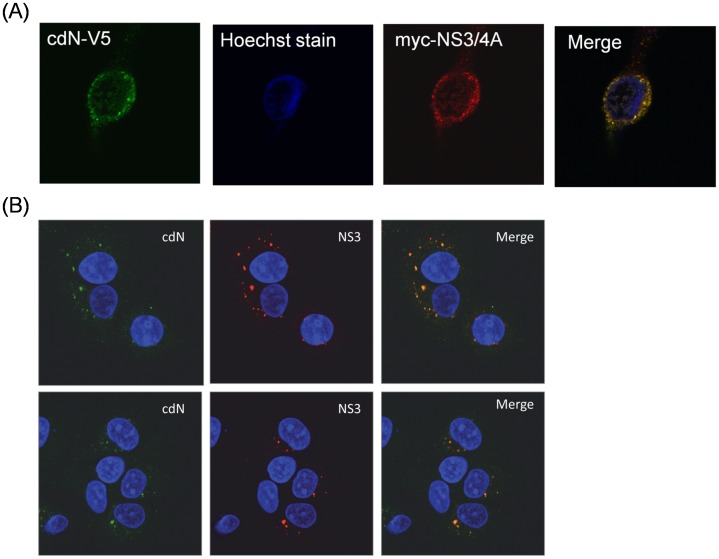
Confocal microscopy analysis of HCV NS3 and cdN proteins in cultured cells. (A) HuH7 cells were co-transfected with the plasmid expressing the cdN protein with a V5 tag and the plasmid expressing HCV NS3/4A protein with a myc tag. After transfection, the cells were fixed and initially stained with mouse anti-myc and Cy3-conjugated anti-mouse antibodies. FITC-conjugated anti-V5 antibodies were then used for staining. (B) HCV subgenomic RNA replicon cells were fixed and stained with mouse anti-NS3 and goat anti-cdN, followed by Cy3-conjugated anti-mouse IgG and FITC-conjugated anti-goat IgG antibodies. Green represents cdN protein; red represents HCV NS3; blue represents nucleus staining with DAPI; the orange color in merged image indicated colocalization of cdN and HCV NS3 proteins.

If NS3 and cdN could indeed bind to each other in cells, then they would likely be co-localized in cells. We had also performed the confocal microscopy analysis to verify the subcellular localization of these two proteins. DNA plasmids expressing the full-length NS3/4A and the cdN proteins were transfected together into HuH7 cells. The cdN protein was found to co-localize with the NS3 protein in the cytoplasm (Fig. 3A). Furthermore, the subcellular localization of these two proteins was examined in HCV sub-genomic replicon cells. Indeed, cdN protein was also found to co-localize with the NS3 protein in the cytoplasm (Fig. 3B).These results indicated that cdN and NS3 could indeed physically interact with each other in cells.

### More than 50% of the 5′(3′)-deoxyribonucleotidase Activity in the HuH7 Cells is from the cdN Protein

**Figure 4:**
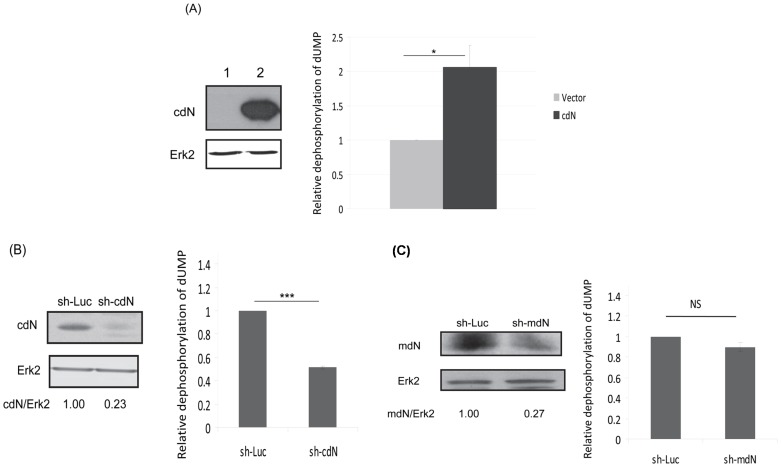
Majority of 5′(3′)-deoxyribonucleotidase activity in the HuH7 cells is from the cdN protein. (A, B) The amount of de-phosphorylation of dUMP correlated with the amount of cdN protein. (A) (Left) HuH7 cells were transfected with empty vector (lane 1) or the cdN plasmid (lane 2). At 48 hrs after transfection, proteins derived from these cells were analyzed using antibodies against V5 tag to detect the exogenous cdN expression (upper panel) or against Erk-2 as a loading control (bottom panel). (Right) The 5′(3′)-deoxyribonucleotidase activity was determined by measuring the relative amount of de-phosphorylation of dUMP. (B) (Left) HuH 7 cells were transduced with lentiviral vector expressing shLuc or a shRNA targeting cdN. After puromycin selection, proteins derived from these cells were analyzed by Western blotting using antibodies against cdN protein to determine the knockdown efficiency (upper panel) or against Erk-2 as a loading control (bottom panel). (Right) The results of 5′(3′)-deoxyribonucleotidase activity assay. (C) The mdN protein was not the major contributor for 5′(3′)-deoxyribonucleotidase activity by measuring the relative level of the de-phosphorylation of dUMP in HuH7 cells. (Left) HuH 7 cells were transduced with lentiviral vector expressing shLuc or the shRNA targeting mdN. After puromycin selection, proteins derived from these cells were analyzed by Western blotting using antibodies against mdN protein to determine the knockdown efficiency (upper panel) or against Erk-2 as a loading control (bottom panel). (Right) The results of 5′(3′)-deoxyribonucleotidase activity assay.

To evaluate the effect of HCV on the enzymatic activity of cdN, the 5′(3′)-deoxyribonucleotidase activity assay to detect the dephosphorylation of dUMP was established following a published procedure [15]. To confirm whether this assay could indeed measure cdN activity, cdN protein was over-expressed exogenously in HuH7 cells as a gain-of-function control. As expected, 5′(3′)-deoxyribonucleotidase activity increased about 2 fold in these cdN protein over-expressed cells (Fig. 4A). To further confirm the accuracy of this assay, shRNAs targeting cdN gene were used as a loss-of-function control. Comparing the control shRNA targeting the luciferase gene, the cdN shRNA reduced the 5′(3′)-deoxyribonucleotidase activity significantly (Fig. 4B). Thus, the 5′(3′)-deoxyribonucleotidase activity assay used in this study could indeed measure the cdN activity.

cdN and mdN (mitochondrial 5′(3′)-deoxyribonucleotidase; dNT-2) catalyze the dephosphorylation of deoxyribonucleoside monophosphates and regulate dTTP formation in cytosol and mitochondria respectively, protecting DNA replication from imbalanced precursor pools [19], [20], [21], [22]. The assay analyzing the dUMP dephosphorylation measures both cdN and mdN activities [15]. To determine the individual contribution of these two proteins in the analysis, shRNA knockdown experiments were performed. When cdN expression was reduced to about 23% by shRNA, the dephosphorylation of dUMP was reduced to about 50% (Fig. 4B). On the other hand, when mdN expression was knocked-down to 27% by shRNA, the dephosphorylation of dUMP was only reduced to 89% (Fig. 4C). Thus, at least 50% of the 5′(3′)-deoxyribonucleotidase activity in the HuH7 cells measured in this assay is derived from cdN protein.

### The Cellular cdN Activity was Partially Repressed by HCV NS3/4A Protein in Both Transiently-transfected and Stably-transfected Systems

**Figure 5:**
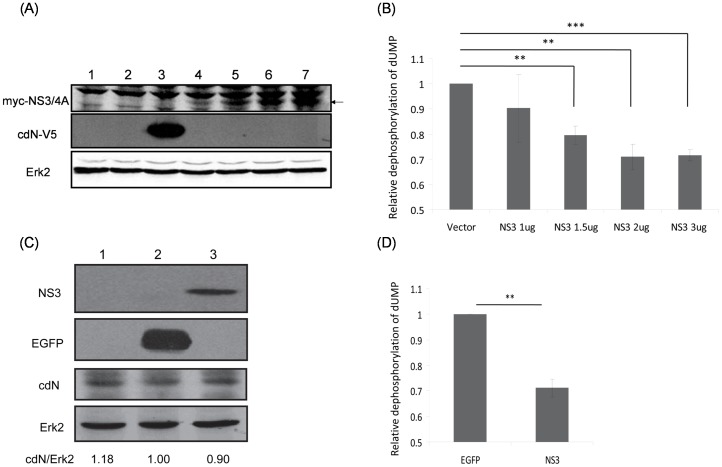
HCV NS3 protein partially represses cellular cdN activity. (A) HuH7 cells were mock-transfected (lane 1) or transfected with empty vector (3 ug, lane 2), the cdN plasmid (3 ug, lane 3) or different amount of myc-NS3/4A plasmids (1 ug, lane 4; 1.5 ug, lane 5; 2 ug, lane 6; 3 ug, lane 7) together with empty vectors to a total of 3 ug DNA in each experiment. At 48 hrs after transfection, proteins derived from these cells were analyzed using antibodies against myc tag to detect the expression of exogenous NS3/4A protein (upper panel), against V5 tag to detect the exogenous cdN expression (middle panel) or against Erk-2 as a loading control (bottom panel). (B) The 5′(3′)-deoxyribonucleotidase activity was measured using cell lysates derived from (A). (C) HuH7 cells were mock-transduced (lane 1) or transduced with lentiviral vectors expressing EGFP (lane 2) or HCV NS3/4A protein (lane 3). After puromycin selection, proteins derived from these cells were analyzed using antibodies against NS3 (upper panel), against EGFP, against cdN protein or against Erk-2 as a loading control (bottom panel). (D) The 5′(3′)-deoxyribonucleotidase activity was analyzed using cell lysates derived from (C).

To determine whether HCV NS3 protein affects the cdN activity since these two proteins interact with each other, plasmids encoding HCV NS3/4A protein were transiently transfected into HuH7 cells (Fig. 5A). The 5′(3′)-deoxyribonucleotidase activity in the HuH7 cells was repressed by NS3/4A protein in a dose dependent manner (Fig. 5B). In this assay, the cells with over-expressed cdN protein were served as a positive control (Fig. 5A). As expected, the 5′(3′)-deoxyribonucleotidase activity measured in these HuH7 cells was about 2 fold of the control (data not shown). HuH7 cells with stable HCV NS3/4A protein expression was also established (Fig. 5C), compared with the HuH7 cells with stable EGFP protein expression, the 5′(3′)-deoxyribonucleotidase activity was repressed to 70% by NS3/4A protein (Fig. 5D) while the amount of cdN protein was not altered significantly (10% reduction, Fig. 5C).

### HCV Partially Represses the cdN Activity while has No Effect on cdN Protein Expression in Both HCV Sub-genomic Replicon Cells and the Infectious HCV Virions Infected Cells

**Figure 6:**
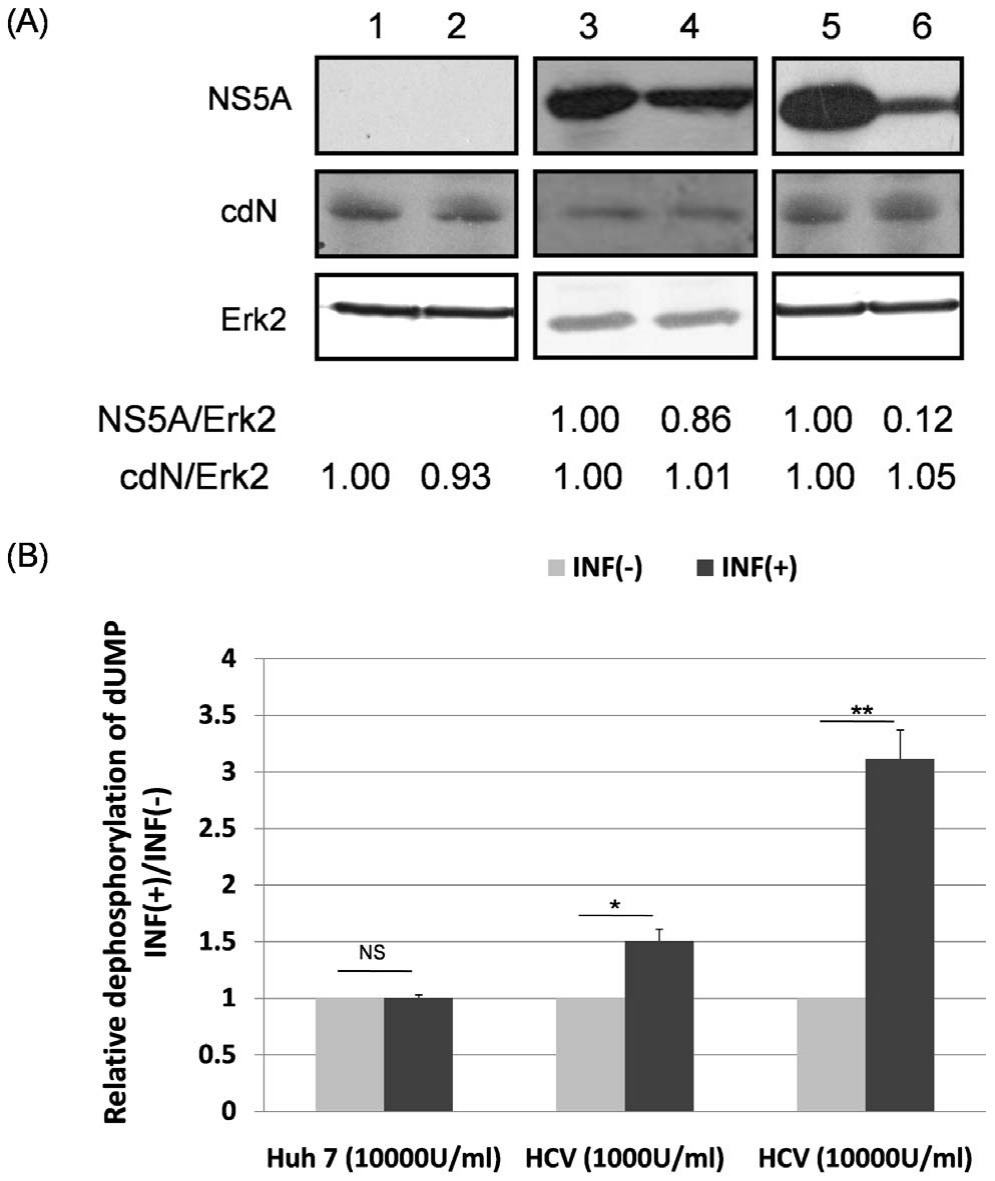
The cdN activity but not its protein amount was increased after interferon treatment in HCV replicon cells. (A) HuH7 cells (lanes 1 and 2) or HCV replicon cells (lanes 3-6) were mock-treated (lanes 1, 3 and 5) or treated with interferon-α (10^4^ U/ml in lanes 2 and 6; 10^3^ U/ml in lane 4). At 72 hrs after treatment, proteins derived from these cells were analyzed using antibodies against NS5A to reflect HCV replication (upper panel), against cdN protein (middle panel) or against Erk-2 as a loading control (bottom panel). (B) The 5′(3′)-deoxyribonucleotidase activity was analyzed using cell lysates derived from (A).

To determine whether HCV infection would affect the host cdN activity, HCV sub-genomic RNA replicon cells were treated with interferon to remove the replicons. As expected, HCV sub-genomic RNA replicons were reduced significantly and dose-dependently as determined by the amount of NS5A protein after the treatment of different amount of interferon (lanes 3-6, top panels of Fig. 6A). The NS5A protein amount was also used to reflect the NS3 protein amount since the expression of these two proteins correlates very well in HCV replicon cells [23]. The 5′(3′)-deoxyribonucleotidase activity was 1.5-fold higher in 10^3^ U/ml interferon treated replicon cells than that of non-treated cells (Fig. 6B) while the amount of cdN protein remained almost the same (Fig. 6A). Moreover, the 5′(3′)-deoxyribonucleotidase activity was three fold higher in 10^4^ U/ml-interferon treated replicon cells than that of non-treated cells (Fig. 6B) while the amount of cdN protein remained at the same level (Fig. 6A). On the other hand, neither the amount of cdN protein nor the 5′(3′)-deoxyribonucleotidase activity in HuH7 cells showed significant changes with or without 10^4^ U/ml interferon treatments (Fig. 6A and 6B).

**Figure 7:**
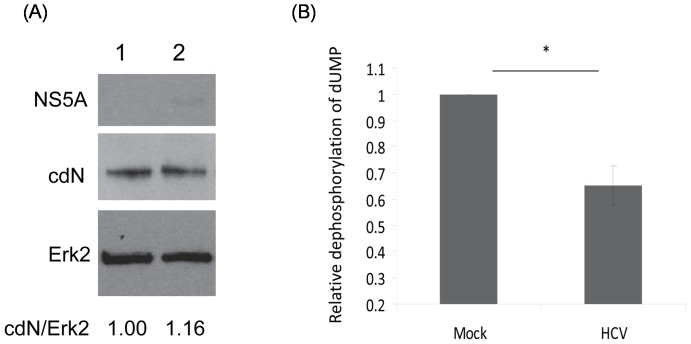
The cdN activity but not its protein amount was partially suppressed by the infection of HCV virions. (A) HuH7.5 cells either mock-infected (lane 1) or infected with 2×10^4^ HCV infectious particles (lane 2). Three days after infection, proteins derived from these cells were analyzed using antibodies against NS5A (upper panel), cdN (middle panel) or against Erk2 as a loading control (bottom panel). (B) The 5′(3′)-deoxyribonucleotidase activity was analyzed using cell lysates derived from (A).

The effect of HCV on cdN activity was also determined in a HCV infectious system [16], [17], [18]. Compared with that of mock-infected HuH7.5 cells, the 5′(3′)-deoxyribonucleotidase activity was reduced significantly in cells infected with infectious HCV virions (Fig. 7B) while the amount of cdN protein was not altered significantly (1.00 vs 1.16, Fig. 7A).

### Cellular cdN Protein did not Affect HCV Replication

**Figure 8:**
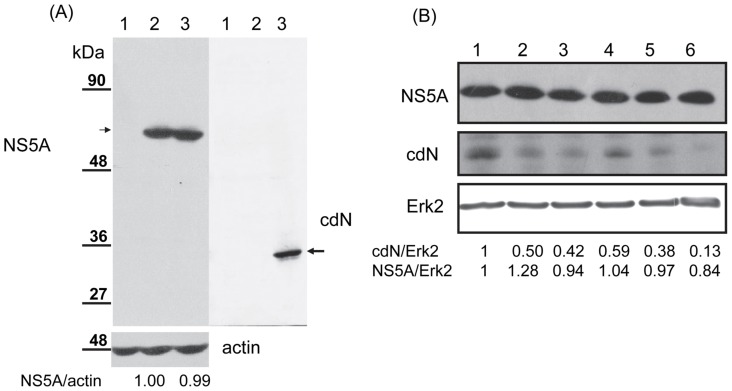
Cellular cdN protein did not affect HCV replication. (A) HCV replicon cells were transfected with empty vector (lane 2) or the plasmid expressing cdN protein with a V5 tag (lane 3). At 48 hrs after transfection, proteins derived from these cells were analyzed by Western blotting using antibodies against NS5A to reflect HCV replication (upper left panel), against V5 to detect cdN expression (right panel) or against actin as a loading control (bottom left panel). Proteins derived from mock-transfected HuH7 cells (lane 1) were served as a negative control for the detection of NS5A. (B) HCV replicon cells were transduced with lentiviral vectors expressing shLuc (lane 1) or different shRNAs targeting cdN gene (lanes 2-6). After puromycin selection, proteins derived from these cells were analyzed by Western blotting using antibodies against NS5A to reflect HCV replication (upper panel), against cdN protein to determine the knockdown efficiency (middle panel) or against Erk-2 as a loading control (bottom panel).

To evaluate the effect of cdN proteins on HCV replication, cdN protein was over-expressed exogenously in the HCV subgenomic cells (Fig. 8A). If cdN protein modulates the NS3 protease activity and, in turn, affects HCV replication, the amount of HCV NS5A protein would be altered in cells with over-expressed cdN protein [23]. However, the amount of HCV NS5A protein did not change in these cells (left panel, Fig. 8A). On the other hand, cdN protein was probably not cleaved by NS3 protein because no potentially cleaved product of cdN was detected in these cells (right panel, Fig. 8A). To further evaluate the effect of cdN proteins on HCV replication, cdN expression was knocked-down in HCV sub-genomic replicon cells. As expected, the cellular cdN protein was reduced to 13%–59% by different shRNAs targeting cdN (middle panel, Fig. 8B). However, the amount of HCV NS5A protein was not altered in these cells (from 0.84 to 1.28, Fig. 8B). Thus, HCV replication was not affected by cdN protein.

## Discussion

In this study, cellular cdN protein was found to interact with NCV NS3 protein in the yeast two-hybrid system (Fig. 1) and in cultured cells (Figs. 2 and 3). This interaction results in the partial suppression of cdN activity by NS3 protein (Fig. 5). Moreover, the cdN activity was also partially repressed by HCV in the systems of HCV sub-genomic replicons and infectious HCV virions (Figs. 6–7).

DNA replication and repair requires a balanced supply of four deoxyribonucleotides (dNTPs) [24]. The intracellular dNTPs pools in mammalian cells are regulated by substrate cycles. Substrate cycles depend on the interplay between a deoxynucleoside kinase and a nucleotidase [25], [26]. Nucleoside monophosphate phosphohydrases or 5′-nucleotidases dephosphorylate non-cyclic nucleoside monophosphate to nucleosides and inorganic phosphates. At least seven 5′-nucleotidases with different subcellular localization have been cloned [19]. Some 5′-nucleotidases are ubiquitous (eN, cN-II, cdN, and mdN) while others display tissue-specific expression (cN-I and cN-III). All 5′-nucleotidases have relatively broad substrate specificities. Detection of individual nucleotidases by enzymatic assays in cell lysates is problematic because different nucleotidases are co-expressed in the same tissue or cell type. cdN is first purified to homogeneity from human placenta [6]. Our results (Fig. 4) showed that cdN contributed more than mdN to the total cellular 5′(3′)-deoxyribonucleotidase activity, which is consistent to previous reports demonstrating that cdN is responsible for the major 5′(3′)-deoxyribonucleotidase activity in cultured human cells [15], [27].

Our results showed that HCV NS3 protein caused a 30% reduction of the cellular 5′(3′)-deoxyribonucleotidase activity in both transiently and stably expressed systems but it did not repress the expression of the cdN protein (Fig. 5). The apparent lowered 5′(3′)-deoxyribonucleotidase activity should be due to a reduction of cdN but not mdN activity for the following reasons: (i) NS3 protein binds cdN (Figs 1–3); (ii) mdN does not co-localize with NS3 protein although it is highly homologous to cdN (52% amino acid identity); (iii) mdN activity is lower in HuH7 cells (Fig. 4C).

The presence of at least seven genes for 5′-nucleotidases in human genome suggests that these enzymes perform important metabolic functions [19]. HCV NS3 protein is involved in cell transformation. The serine protease domain of NS3 protein is responsible for the cell transformation [4], [5]. HCV NS3 protein derived from certain genotypes has been demonstrated to generate an internally cleaved product containing the protease domain [28], [29]. Our results demonstrated that HCV NS3 protein interacts with cellular cdN protein (Figs 1 to 3), and the protease domain of NS3 was mapped for this interaction. Therefore, our results suggested that the pathogenesis induced by NS3 or its internally cleaved product may be mediated by binding to cdN protein and inhibiting its activity.

In HCV replicon cells, our results showed that the 5′(3′)-deoxyribonucleotidase activity measured after 10^4^ U/ml interferon treatment was three fold higher than that of the non-treated cells while the amount of cdN protein remained almost the same (Fig. 6). We further showed that HCV repressed the 5′(3′)-deoxyribonucleotidase activity to 65% but did not affect the level of cdN protein expression (Fig. 7). Therefore, we concluded that HCV down-regulated cdN activity, and this inhibitory effect is more pronounced in HCV replicons than in HCV infected cells due to the greater amount of NS3 protein in HCV replicon cells. Thus, attenuating cellular 5′(3′)-deoxyribonucleotidase activity may be a strategy for HCV to benefit its replication. However, neither over-expression nor knockdown of cdN gene affected HCV replication (Fig. 8). The biologic meaning of attenuating cellular 5′(3′)-deoxyribonucleotidase activity in HCV-infected cells awaits further investigation. Interestingly, the ecto-5′nucleotidase activity in B lymphocytes was low in HIV-infected patients [30].

Control of DNA precursor concentrations is essential for the maintenance of genetic stability because DNA precursor pool imbalances can elicit a variety of genetic effects [31], [32]. cdN, a ubiquitous dNT enzyme, is important for the control of intracellular dNTPs pools [19]. HCV infection is etiologically involved in the development of hepatocellular carcinoma and B-cell lymphomas [2], [3]. It is likely that HCV induces tumor cell formation through the partial inhibition of cdN activity by NS3 protein to result in the imbalance of DNA precursor concentrations.

In summary, HCV NS3 protein was found to interact with cellular cdN protein, and, in turn partially inhibits its activity. HCV may induce diseases through inhibition of cdN activity by NS3 protein.

## References

[1] Chien DY, Choo QL, Tabrizi A, Kuo C, McFarland J, et al (1992) Diagnosis of hepatitis C virus (HCV) infection using an immunodominant chimeric polyprotein to capture circulating antibodies: reevaluation of the role of HCV in liver disease. Proc Natl Acad Sci U S A 89: 10011–10015. 1279666 10.1073/pnas.89.21.10011 PMC50267

[2] Negri E, Little D, Boiocchi M, La Vecchia C, Franceschi S (2004) B-cell non-Hodgkin’s lymphoma and hepatitis C virus infection: a systematic review. Int J Cancer 111: 1–8. 15185336 10.1002/ijc.20205

[3] Lemon SM, Walker CM, Alter MJ, and Yi M (2007) Hepatitis C Virus. In: D. M. Knipe aH, P.M., editor. Fields’ Virology,. Fifth ed. Philadelphia: Lippincott Williams & Wilkins,. 1253–1304.

[4] Zemel R, Gerechet S, Greif H, Bachmatove L, Birk Y, et al (2001) Cell transformation induced by hepatitis C virus NS3 serine protease. J Viral Hepat 8: 96–102. 11264729 10.1046/j.1365-2893.2001.00283.x

[5] Sakamuro D, Furukawa T, Takegami T (1995) Hepatitis C virus nonstructural protein NS3 transforms NIH 3T3 cells. J Virol 69: 3893–3896. 7745741 10.1128/jvi.69.6.3893-3896.1995 PMC189112

[6] Hoglund L, Reichard P (1990) Cytoplasmic 5′(3′)-nucleotidase from human placenta. J Biol Chem 265: 6589–6595. 2157703

[7] Rampazzo C, Johansson M, Gallinaro L, Ferraro P, Hellman U, et al (2000) Mammalian 5′(3′)-deoxyribonucleotidase, cDNA cloning, and overexpression of the enzyme in Escherichia coli and mammalian cells. J Biol Chem 275: 5409–5415. 10681516 10.1074/jbc.275.8.5409

[8] Ma HC, Ku YY, Hsieh YC, Lo SY (2007) Characterization of the cleavage of signal peptide at the C-terminus of hepatitis C virus core protein by signal peptide peptidase. J Biomed Sci 14: 31–41. 17237979 10.1007/s11373-006-9127-1 PMC7088784

[9] Skapek SX, Jansen D, Wei TF, McDermott T, Huang W, et al (2000) Cloning and characterization of a novel Kruppel-associated box family transcriptional repressor that interacts with the retinoblastoma gene product, RB. J Biol Chem 275: 7212–7223. 10702291 10.1074/jbc.275.10.7212

[10] Hidajat R, Nagano-Fujii M, Deng L, Tanaka M, Takigawa Y, et al (2005) Hepatitis C virus NS3 protein interacts with ELKS-{delta} and ELKS-{alpha}, members of a novel protein family involved in intracellular transport and secretory pathways. J Gen Virol 86: 2197–2208. 16033967 10.1099/vir.0.80862-0

[11] Wei WY, Li HC, Chen CY, Yang CH, Lee SK, et al (2012) SARS-CoV nucleocapsid protein interacts with cellular pyruvate kinase protein and inhibits its activity. Arch Virol 157: 635–645. 22222284 10.1007/s00705-011-1221-7 PMC7087308

[12] Yang CH, Li HC, Jiang JG, Hsu CF, Wang YJ, et al (2010) Enterovirus type 71 2A protease functions as a transcriptional activator in yeast. J Biomed Sci 17: 65. 20682079 10.1186/1423-0127-17-65 PMC2923119

[13] Ma HC, Lin TW, Li H, Iguchi-Ariga SM, Ariga H, et al (2008) Hepatitis C virus ARFP/F protein interacts with cellular MM-1 protein and enhances the gene trans-activation activity of c-Myc. J Biomed Sci 15: 417–425. 18398700 10.1007/s11373-008-9248-9

[14] Choi J, Lee KJ, Zheng Y, Yamaga AK, Lai MM, et al (2004) Reactive oxygen species suppress hepatitis C virus RNA replication in human hepatoma cells. Hepatology 39: 81–89. 14752826 10.1002/hep.20001

[15] Rampazzo C, Mazzon C, Reichard P, Bianchi V (2002) 5′-Nucleotidases: specific assays for five different enzymes in cell extracts. Biochem Biophys Res Commun 293: 258–263. 12054593 10.1016/S0006-291X(02)00206-1

[16] Lindenbach BD, Evans MJ, Syder AJ, Wolk B, Tellinghuisen TL, et al (2005) Complete replication of hepatitis C virus in cell culture. Science 309: 623–626. 15947137 10.1126/science.1114016

[17] Wang SY, Tseng CP, Tsai KC, Lin CF, Wen CY, et al (2009) Bioactivity-guided screening identifies pheophytin a as a potent anti-hepatitis C virus compound from Lonicera hypoglauca Miq. Biochem Biophys Res Commun 385: 230–235. 19450556 10.1016/j.bbrc.2009.05.043

[18] Cheng JC, Yeh YJ, Tseng CP, Hsu SD, Chang YL, et al (2012) Let-7b is a novel regulator of hepatitis C virus replication. Cell Mol Life Sci 69: 2621–2633. 22391672 10.1007/s00018-012-0940-6 PMC11115169

[19] Bianchi V, Spychala J (2003) Mammalian 5′-nucleotidases. J Biol Chem 278: 46195–46198. 12947102 10.1074/jbc.R300032200

[20] Wallden K, Rinaldo-Matthis A, Ruzzenente B, Rampazzo C, Bianchi V, et al (2007) Crystal structures of human and murine deoxyribonucleotidases: insights into recognition of substrates and nucleotide analogues. Biochemistry 46: 13809–13818. 17985935 10.1021/bi7014794

[21] Rampazzo C, Gallinaro L, Milanesi E, Frigimelica E, Reichard P, et al (2000) A deoxyribonucleotidase in mitochondria: involvement in regulation of dNTP pools and possible link to genetic disease. Proc Natl Acad Sci U S A 97: 8239–8244. 10899995 10.1073/pnas.97.15.8239 PMC26931

[22] Mazzon C, Rampazzo C, Scaini MC, Gallinaro L, Karlsson A, et al (2003) Cytosolic and mitochondrial deoxyribonucleotidases: activity with substrate analogs, inhibitors and implications for therapy. Biochem Pharmacol 66: 471–479. 12907246 10.1016/s0006-2952(03)00290-9

[23] Chan SC, Lo SY, Liou JW, Lin MC, Syu CL, et al (2011) Visualization of the structures of the hepatitis C virus replication complex. Biochem Biophys Res Commun 404: 574–578. 21147066 10.1016/j.bbrc.2010.12.037

[24] Reichard P (1988) Interactions between deoxyribonucleotide and DNA synthesis. Annu Rev Biochem 57: 349–374. 3052277 10.1146/annurev.bi.57.070188.002025

[25] Hunsucker SA, Mitchell BS, Spychala J (2005) The 5′-nucleotidases as regulators of nucleotide and drug metabolism. Pharmacol Ther 107: 1–30. 15963349 10.1016/j.pharmthera.2005.01.003

[26] Hoglund L, Pontis E, Reichard P (1988) Effects of deoxycytidine and thymidine kinase deficiency on substrate cycles between deoxyribonucleosides and their 5′-phosphates. Cancer Res 48: 3681–3687. 2837322

[27] Gallinaro L, Crovatto K, Rampazzo C, Pontarin G, Ferraro P, et al (2002) Human mitochondrial 5′-deoxyribonucleotidase. Overproduction in cultured cells and functional aspects. J Biol Chem 277: 35080–35087. 12124385 10.1074/jbc.M203755200

[28] Shoji I, Suzuki T, Sato M, Aizaki H, Chiba T, et al (1999) Internal processing of hepatitis C virus NS3 protein. Virology 254: 315–323. 9986797 10.1006/viro.1998.9540

[29] Kou YH, Chang MF, Wang YM, Hung TM, Chang SC (2007) Differential requirements of NS4A for internal NS3 cleavage and polyprotein processing of hepatitis C virus. J Virol 81: 7999–8008. 17522200 10.1128/JVI.00348-07 PMC1951333

[30] Dalh Christensen L, Svenson M, Nygaard P, Andersen V, Faber V (1988) Decreased B lymphocyte ecto-5′nucleotidase and increased adenosine deaminase in mononuclear cells from patients infected with human immunodeficiency virus. APMIS 96: 882–888. 2847768 10.1111/j.1699-0463.1988.tb00955.x

[31] Kunz BA (1988) Mutagenesis and deoxyribonucleotide pool imbalance. Mutat Res 200: 133–147. 3292903 10.1016/0027-5107(88)90076-0

[32] Kunz BA, Kohalmi SE, Kunkel TA, Mathews CK, McIntosh EM, et al (1994) International Commission for Protection Against Environmental Mutagens and Carcinogens. Deoxyribonucleoside triphosphate levels: a critical factor in the maintenance of genetic stability. Mutat Res 318: 1–64. 7519315 10.1016/0165-1110(94)90006-x

